# A modified dental age assessment method for 5- to 16-year-old eastern Chinese children

**DOI:** 10.1007/s00784-020-03668-9

**Published:** 2021-01-09

**Authors:** Jing Pan, Checheng Shen, Zhao Yang, Linfeng Fan, Miaochen Wang, Shihui Shen, Jiang Tao, Fang Ji

**Affiliations:** 1 Department of Orthodontics, Ninth People’s Hospital, Shanghai Jiao Tong University School of Medicine, Shanghai, China; 2 Department of General Dentistry, Ninth People’s Hospital, Shanghai Jiao Tong University School of Medicine, Shanghai, China

**Keywords:** Chinese children, Polynomial curve fitting, Modified dental age estimation, Demirjian method, Willems method

## Abstract

**Objectives:**

Age estimation is widely applied in the field of orthodontics, pediatric dentistry, and forensic science. Dental age estimation by the radiological method is frequently used because of its convenience and noninvasiveness. However, there are not enough suitable methods for eastern Chinese children. This study aimed to establish a modified formula for eastern Chinese children according to the Demirjian method and then compared the accuracy of the modified method with the Demirjian method and Willems method.

**Materials and methods:**

A total of 2367 dental panoramic radiographs from individuals aged 5–16 years of eastern China were collected as samples. Age estimation was conducted using the Demirjian and Willems methods. The polynomial curve fitting method was used to modify the Demirjian method to improve its application to the eastern Chinese children. The paired *t* test and accuracy ratio were used to compare the applicability of the modified methods with two commonly used methods.

**Results:**

The mean chronological age (CA) of the subjects was 11.20 ± 3.29 years for boys and 10.99 ± 3.12 years for girls. The mean difference values between the CA and dental age (DA) (CA–DA) using the Demirjian and Willems methods were 0.73 and 0.7 for boys, respectively, and both 0.79 for girls. The modified method using the polynomial curve fitting presented a smaller underestimation compared with CA for both boys (0.04 years) and girls (0.09 years), which showed a high suitability to Chinese children to some extent.

**Conclusions:**

The Willems method was more accurate in estimating DA compared with the Demirjian method. However, the modified method was more accurate than the two methods; therefore, it can be used in eastern Chinese children.

**Clinical relevance:**

It was thought to be a non-invasive, convenient, and efficient method to connect DA and CA. By estimating dental age, pediatrist, and orthodontists can better understand the development of permanent teeth and provide a more accurate orthodontic treatment time and treatment plan to children patients.

## Introduction

Age estimation has been frequently applied in the field of orthodontics, pediatric dentistry, anthropology, and archaeology [1, 2]. In circumstances wherein the actual age is ambiguous, it is usually used to assess the profile and age of a person [3, 4]. Age estimation is mainly used to determine certain social responsibilities, such as criminal responsibility, legal ability, and employment, marriage, or immigration responsibility [3–6]. Moreover, assessment of the age is also used in the treatment plan and diagnostic tools in orthodontics and pediatric dentistry [7–9].

To date, there are various methods to estimate the chronological age (CA), including clinical findings like fusion of sterno-clavicular bones, molecular biomarkers such as DNA methylation, left hand imaging, and tooth age assessment. Compared with skeletal development indicators [10], teeth can be used as a more reliable indicator of maturity, because it is controlled by genes and is independent of the individual’s somatic growth [11]. The degree of tooth mineralization is less affected by mechanical or chemical corrosions and is virtually immune to physical factors such as lack of deciduous teeth, insufficient space, dental caries, and some orthodontic deformities [2, 10, 11].

The radiological method proposed by Demirjian et al. [12] in 1973 in the study of French-Canadian individuals is the most widely applied method in dental age (DA) assessment at present, due to its rationality, ease of application, and objectivity [3].

The age prediction performance of the Demirjian method has been validated in Spain [13], Tunisia [14], France [15, 16], Malaysia [17], and other countries [4, 10, 16, 18], leading to an overestimation of CA. The Willems method simplifies the transformation steps of the Demirjian method, creating a new table. It showed that, as tested in different populations, including Somali [16], South Australian [18], Navi Mumbai [19], and Malaysian [20], the accuracy of estimating DA was consistently higher than that of the Demirjian method [21, 22]. Ethnic differences are thought to be a significant factor in the development of dental maturity [23–25]. For example, the DA by the Demirjian method for Chinese northern children comparing with the Willems method obviously overestimates the dental of boys and girls, which is not accurate [26]. However, according to the estimation of the DA of children in northern China, the Demirjian method underestimated the age of children in northern China, similar to Willems method [27]. Due to the fact that ethnicity is dependent on economic, political, and nutrition, it is clear that one set of dental age estimation method does not fit all populations including Chinese eastern population. Therefore, this study aimed to establish a modified formula to assess dental age in eastern Chinese children based on an adaptation of the Demirjian method and then compared the accuracy of the modified method with the Demirjian method and Willems method.

## Materials and methods

### Sample

In this retrospective study, 2367 samples were derived from patients who underwent treatment in Shanghai Ninth People’s Hospital affiliated with Shanghai Jiao Tong University, School of Medicine. The patients were all from eastern China, including Jiangsu, Zhejiang, and Shanghai province, which all have the same eating habits, geographical environment, and economic development level. The dental panoramic radiographs were obtained from 1217 boys and 1150 girls, with age ranging from 5 to 16 years. Radiographs were collected from the hospital information sheet from October 1, 2016 to March 31, 2018, and the birth dates of these children were from January 1, 2000 to February 28, 2013. These data were divided into 12 age groups every other year. The ethics approval for this study was granted by the Independent Ethics Committee of the Shanghai Ninth People’s Hospital affiliated with Shanghai Jiao Tong University, School of Medicine.

The inclusion criteria were as follows:

1. Han ethnic origin.
2. Age between 5 and 16 years.
3. Panoramic radiographs with clear and high-quality images and no obvious development pathology.
4. Complete dental records of radiographs, including the date of birth, date of radiography, and sex.

The exclusion criteria were as follows:

1. Bilateral absence of the teeth in the mandible (except the third molar).
2. Malocclusion or maxillofacial abnormalities.
3. Previous orthodontic or endodontic treatment.
4. Systemic disease or history of dental trauma.
5. Shape and position anomalies.
6. Any existing pathological condition relevant to the jaw bone, such as cysts or tumors.
7. Congenital and genetic anomalies.
8. The radiographs were of poor quality or blurred, and patient records were incomplete.

## Materials and methods

The CA of each subject was calculated by subtracting the date of birth from the date of panoramic radiography, and the results were expressed as years with two decimal places.

### Dental age estimation by the Demirjian method and Willems method

The dental age estimation was performed based on the maturity of the seven permanent teeth on the left mandible in the radiographs. According to the Demirjian staging criterion [12], tooth developments were classified into eight distinct stages, which were identified by letters “A” to “H”. The designated stages started with initial crown formation and continued until closure of the root apex. Each tooth was rated on a scale, and each rating was then converted into a score according to the table provided by Demirjian. The DA was obtained from the total maturity score of the seven teeth as described by Demirjian [12, 28]. The Willems method is a simplified process of the Demirjian method [29]. After converting each rating to a score according to the Willems method, the DA was obtained by adding the scores of the seven teeth.

### Modified dental estimation

After testing the Demirjian and Willems methods in the eastern Chinese children, when paired *t* test showed the significant difference between DA and CA, a mathematical model was constructed to modify the Demirjian method using the polynomial curve fitting method (PCFM) to estimate the relationship between the Demirjian maturity score and CA, so as to obtain more accurate results. Since the Willems method was modified from the Demirjian method (2001), the score obtained from the Demirjian method was adopted to establish a more suitable scale for the Chinese population.

PCFM was used to find the coefficients of CA of degree *n* that fits the maturity score where the degree *n* can be selected according to the requirement of errors. It can fit a sufficient number of data points, which had good predictive power for both known and unknown data. It matched by minimizing the square of the error and finding the best function for the data.

The form of it is as follows:

$$ \mathrm{p}\left(\mathrm{x}\right)={\mathrm{p}}_1{\mathrm{x}}^{\mathrm{n}}+{\mathrm{p}}_2{\mathrm{x}}^{\mathrm{n}-1}+\dots +{\mathrm{p}}_{\mathrm{n}}\mathrm{x}+{\mathrm{p}}_{\mathrm{n}+1} $$

### Inter-examiner and intra-examiner reliability

The digital orthopantomograms were scored separately by three observers who were skilled in both Demirjian and Willems methods. To avoid bias, they all had no information on patients’ details, such as name, age, or sex. One hundred randomly selected radiographs were scored by the observers after 2 weeks. The inter- and intra-rater agreement was assessed using Cohen’s kappa analysis.

### Statistical methods

The statistical data were analyzed using Statistical Analysis System (SAS 9.4) software, and data management was performed using Microsoft Excel 2010. The relationship between the CA and DA was analyzed for each sex and age group. Besides calculation of the mean and standard deviation of the CA and DA, the difference between the CA and DA was tested using paired Student’s *t* test at a significance level of *p* < 0.05. Moreover, we usually use integers for age, so we assumed that, when |CA-DA| ≤ 1, the result was applicative and meaningful; then, we calculated the number and percentage of accuracy within this specific time interval.

## Results

Statistical analysis revealed no statistically significant intra- and inter-observer differences, with kappa coefficients of 0.756 and 0.728, respectively. The mean CAs of boys and girls were basically equal, which were 11.20 ± 3.29 years and 10.99 ± 3.12 years, respectively. The distribution of the 2367 samples by age and sex is shown in Table 1.

**Table 1:** Distribution of samples by age and sex

Sex/Age	Male	Female	Total
5.00-5.99	40	46	86
6.00-6.99	126	102	228
7.00-7.99	104	106	210
8.00-8.99	101	112	213
9.00-9.99	97	91	188
10.00-10.99	108	112	220
11.00-11.99	114	115	229
12.00-12.99	112	127	239
13.00-13.99	109	93	202
14.00-14.99	103	97	200
15.00-1599	109	944	203
16.00-16.99	94	55	149
Total	1217	1150	2367

### Comparison between chronological age and dental age

The difference between chronological age (CA) and dental age (DA) were calculated independently for boys and girls. The descriptive statistics of the age subtractions (CA-DA) using the Demirjian and Willems methods are presented in Tables 2 and 3, respectively.

**Table 2:** Differences between mean chronological age (CA) and calculated dental age (DA) using the Demirjian method and *p*- values of the differences in various age groups for both sexes

Age	Sex	Mean(SD)			95% CI of age difference	t statistics	p values	MAE
		Chronological age	Demirjian dental age (DDA)	Age difference (CA-DDA)				
5.00-5.99	M	5.73 (0.18)	7.09 (0.42)	-1.36 (0.40)	-1.48; -1.23	-21.74	<0.001	1.36
	F	5.67 (0.26)	6.80 (0.43)	-1.13 (0.40)	-1.24; -1.02	-20.74	<0.001	1.29
6.00-6.99	M	6.47 (0.29)	7.46 (0.42)	-0.99 (0.36)	-1.05; -0.92	-30.32	<0.001	0.99
	F	6.48 (0.26)	7.44 (0.30)	-0.96 (0.32)	-1.03; -0.90	-30.68	<0.001	0.93
7.00-7.99	M	7.50 (0.32)	8.05 (0.47)	-0.56 (0.42)	-0.64; -0.47	-13.40	<0.001	0.58
	F	7.59 (0.28)	7.93 (0.48)	-0.34 (0.47)	-0.44; -0.25	-7.49	<0.001	0.36
8.00-8.99	M	8.39 (0.26)	8.72 (0.51)	-0.33 (0.52)	-0.44; -0.23	-6.42	<0.001	0.46
	F	8.48 (0.29)	8.56 (0.64)	-0.08 (0.57)	-0.18; 0.03	-1.42	0.158	0.57
9.00-9.99	M	9.59 (0.29)	9.51 (0.65)	0.01 (0.63)	-0.11; 0.14	0.25	0.803	0.52
	F	9.45 (0.29)	9.58 (0.74)	-0.13 (0.72)	-0.28; 0.02	-1.73	0.087	0.62
10.00-10.99	M	10.50 (0.30)	10.34 (0.77)	0.15 (0.70)	0.02; 0.29	2.36	0.020	0.6
	F	10.49 (0.29)	10.71 (0.86)	-0.22 (0.79)	-0.37; -0.07	-2.91	0.004	0.71
11.00-11.99	M	11.42 (0.25)	11.35 (1.04)	0.07 (0.95)	-0.10; 0.25	0.80	0.423	0.72
	F	11.55 (0.30)	12.06 (1.08)	-0.50 (0.97)	-0.68; -0.33	-0.50	0.965	0.86
12.00-12.99	M	12.37 (0.28)	12.89 (0.95)	-0.51 (0.93)	-0.69; -0.34	-5.87	<0.001	0.83
	F	12.48 (0.29)	12.86 (0.86)	-0.38 (0.91)	-0.54; 0.22	-4.72	<0.001	0.81
13.00-13.99	M	13.48 (0.30)	13.83 (1.16)	-0.35 (1.12)	-0.56; -0.14	-3.26	<0.001	0.98
	F	13.43 (0.25)	13.05 (1.01)	-0.39 (1.00)	0.18; 0.59	3.72	<0.001	0.93
14.00-14.99	M	14.48 (0.30)	14.58 (1.10)	-0.11 (1.07)	-0.31; 0.10	-1.00	0.317	0.87
	F	14.57 (0.30)	14.07 (0.91)	0.49 (0.89)	0.31; 0.67	0.49	0.888	1.01
15.00-15.99	M	15.48 (0.29)	15.33 (0.74)	0.15 (0.75)	0.00; 0.29	2.08	0.039	0.53
	F	15.37 (0.23)	14.66 (0.76)	0.70 (0.82)	0.53; 0.87	8.38	<0.001	0.69
16.00-16.99	M	16.40 (0.27)	15.78 (0.32)	0.63 (0.38)	0.55; 0.70	0.63	0.385	0.63
	F	16.45 (0.24)	15.14 (0.86)	1.30 (0.86)	1.07; 1.53	11.24	<0.001	1.22

**Table 3:** Differences between mean chronological age (CA) and calculated dental age (DA) using the Williems method and p-values of the differences in various age groups for both sexes

Age	Sex	Chronological age	Mean (SD)		95% CI of age difference	t statistics	p values	MAE
			Willems age (WDA)	Age difference (CA-WDA)				
5.00-5.99	M	5.73 (0.18)	6.40 (0.61)	-0.67 (0.58)	-0.85; -0.48	-7.20	<0.001	0.7
	F	5.67 (0.26)	5.93 (0.44)	-0.26 (0.39)	-0.37; -0.14	-4.52	<0.001	0.65
6.00-6.99	M	6.47 (0.29)	6.99 (0.67)	-0.52 (0.56)	-0.62; -0.42	-10.45	<0.001	0.64
	F	6.48 (0.26)	6.81 (0.53)	-0.33 (0.48)	-0.42; -0.23	-6.93	<0.001	0.49
7.00-7.99	M	7.50 (0.32)	7.87 (0.60)	-0.37 (0.52)	-0.47; -0.27	-7.37	<0.001	0.52
	F	7.59 (0.28)	7.67 (0.61)	-0.08 (0.60)	-0.20; 0.03	-1.43	0.156	0.47
8.00-8.99	M	8.39 (0.26)	8.76 (0.62)	-0.37 (0.60)	-0.49; -0.25	-6.12	<0.001	0.53
	F	8.48 (0.29)	8.47 (0.68)	0.02 (0.63)	-0.10; 0.14	0.02	0.930	0.65
9.00-9.99	M	9.53 (0.29)	9.68 (0.72)	-0.15 (0.70)	-0.29; 0.00	-2.08	0.041	0.6
	F	9.45 (0.29)	9.54 (1.30)	-0.08 (1.34)	-0.36; -0.19	-0.60	0.551	0.79
10.00-10.99	M	10.50 (0.30)	10.54 (0.70)	-0.04 (0.66)	-0.17; 0.07	-0.72	0.470	0.53
	F	10.49 (0.29)	10.43 (0.75)	0.06 (0.67)	-0.07; 0.18	0.90	0.368	0.64
11.00-11.99	M	11.42 (0.25)	11.34 (0.88)	0.08 (0.81)	-0.07; 0.23	1.09	0.277	0.6
	F	11.55 (0.30)	11.63 (0.96)	-0.08 (0.86)	-0.23; 0.08	-0.95	0.342	0.71
12.00-12.99	M	12.37 (0.28)	12.47 (0.81)	-0.10 (0.79)	-0.25; 0.04	-1.33	0.185	0.62
	F	12.48 (0.29)	12.44 (0.83)	0.03 (0.86)	-0.11; 0.18	0.42	0.678	0.66
13.00-13.99	M	13.48 (0.30)	13.30 (1.00)	0.18 (95)	0.00; 0.36	2.02	0.046	0.81
	F	13.43 (0.25)	12.80 (0.96)	0.63 (0.95)	0.44; 0.82	6.39	<0.001	1.03
14.00-14.99	M	14.48 (0.30)	13.97 (1.05)	0.51 (1.02)	0.31; 0.71	5.10	<0.001	0.93
	F	14.57 (0.30)	13.76 (0.91)	0.81 (0.90)	0.62; 0.99	8.80	<0.001	1.21
15.00-15.99	M	15.48 (0.29)	14.70 (0.85)	0.78 (0.85)	0.62; 0.94	9.67	<0.001	0.9
	F	15.37 (0.23)	14.27 (0.84)	1.10 (0.90)	0.92; 1.28	11.91	<0.001	1.03
16.00-16.99	M	16.40 (0.27)	15.35 (0.59)	1.06 (0.60)	0.94; 1.18	17.01	<0.001	1.06
	F	16.45 (0.24)	14.70 (0.95)	1.75 (0.95)	1.49; 2.01	13.67	<0.001	1.68
Total	M	11.20 (3.29)	11.13 (2.91)	0.07 (0.92)	0.02; 0.12	2.69	0.007	0.7
	F	10.99 (3.12)	10.75 (2.79)	0.24 (1.03)	0.18; 0.30	7.96	<0.001	0.79

Table 2 shows that, in the Demirjian method, the most frequently observed age difference was between 0 and 1 year in boys and between 0 and 0.5 year in girls. In boys between 6.00 and 15.99 years of age, the DA yielded an insignificant under- and overestimation compared with the CA, and a significant underestimation is noted in those aged 16 years. In girls, the DA showed a slight overestimation between 6.00 and 13.99 years and underestimation between 14.00 and 16.99 years. The largest validation was 1.36 years in age 5 years in boys and 1.30 years in age 16 years in girls.

Table 3 shows all samples using the Willems method. The most frequently observed difference was 0–0.5 years. The DA presented a slight difference between ages 5.00 and 14.99 years in both sexes and a significant underestimation between ages 15.00 and 16.99 years. The largest under- and overestimation of age was 1.06 years (boys) and 1.75 years (girls).

Using both methods, the most significant validation of CA-DA was 16 years for both sexes. However, the Demirjian method showed more than 1-year bias in individuals aged 5 and 16 years, which resulted in a larger difference compared with the Willems method. The mean absolute error (MAE) using the Willems method for each sex (0.7 years for boys and 0.79 years for girls) was similar with that using the Demirjian method (0.73 year for boys and 0.79 year for girls).

Table 4 shows the percentages of CA-DA values within ± 1 year for both sexes in all age groups. The accuracy using the Demirjian method was 73.9% for boys and 70.2% for girls and 75.0% for boys and 75.9% for girls by using the Willems methods. Between ages 5.00 and 6.99 years, using the Demirjian method, the percentage accuracy varied from 15.0 to 54.0% and was only 38.2% for girls aged 16 years. The Willems method was extremely accurate in ages between 5.00 and 6.99 years (varying from 80.0 to 97.8%), but accuracies varied from 32.7 to 65.1% in those aged between 15.00 and 16.99 years.

**Table 4:** Percentages of accuracy for dental age (DA) estimated using the Demirjian and Willems methods for difference values within 1 year from chronological age (CA) for both sexes

Age	Demirjian method		Willems method	
	Male	Female	Male	Female
5	6 (15.0%)	16(34.8%)	32(38.0%)	45(97.8%)
6	68(54.0%)	54(52.9%)	102(81.0%)	96(94.1%)
7	93(89.4%)	101(95.3%)	95(91.3%)	97(91.5%)
8	90(89.1%)	101(90.2%)	85(84.2%)	99(88.4%)
9	85(87.6%)	74(81.3%)	78(84.2%)	76(83.5%)
10	91(84.3%)	91(81.2%)	93(86.1%)	97(86.6%)
11	81(71.1%)	72(62.6%)	89(78.1%)	89(77.4%)
12	82(72.3%)	83(65.4%)	89(78.1%)	97(76.4%)
13	62(56.9%)	62(66.7%)	68(62.4%)	56(60.2%)
14	63(61.2%)	68(70.1%)	66(64.1%)	57(58.8%)
15	97(89.0%)	64(68.1%)	71(65.1%)	46(48.9%)
16	82 (87.2%)	21(38.2%)	45(47.9%)	18(32.7%)
Total	900(73.9%)	807(70.2%)	913(75.0%)	873(75.9%)

Therefore, the Willems method resulted in a more accurate estimation in lower, relative to higher, age groups in both sexes. The Demirjian method showed better accuracy in ages 8.00–15.99 years. However, in both sexes, the Demirjian method and the Willems method provided quite accuracy in eastern Chinese children.

### Modified dental estimation methods

PCFM was employed to estimate the age based on the maturity score values in our study as follows: (*x* represents maturity score; *p(x)* represents age; *p*_*1*_*-p*_*n+1*_ are all constants)

$$ \mathrm{p}\left(\mathrm{x}\right)={\mathrm{p}}_1{\mathrm{x}}^{\mathrm{n}}+{\mathrm{p}}_2{\mathrm{x}}^{\mathrm{n}-1}+\dots +{\mathrm{p}}_{\mathrm{n}}\mathrm{x}+{\mathrm{p}}_{\mathrm{n}+1} $$

According to the eastern Chinese samples, two formulas are used:

$$ \mathrm{Boys}:p(x)= 0.000000304471040683471\ast {x}^5- 0.000104409502065903\ast {x}^4+ 0.0141677515859983\ast {x}^3- 0.948508480952154\ast {x}^2+ 31.3447464323149\ast x- 403.48344718101 $$

$$ \mathrm{Girls}:p(x)= 0.000000461590755570541\ast {x}^5- 0.000154896783268403\ast {x}^4+ 0.0203841924163241\ast {x}^3- 1.31156808447141\ast {x}^2+ 41.2383716718296\ast x- 501.400716931816 $$

The modified formula was used to establish a new relationship between the maturity score and DA between 5 and 17 years. This resulted in new tables for boys and girls with scores directly converting to age (Tables 5 and 6).

**Table 5:** New table to convert the maturity score to dental age for boys

Age	Score	Age	Score	Age	Score
boys					
5.1	39.92	9.1	83.62	13.1	95.49
5.2	41.56	9.2	84.19	13.2	95.63
5.3	43.18	9.3	84.74	13.3	95.73
5.4	44.77	9.4	85.27	13.4	95.89
5.5	46.33	9.5	85.78	13.5	96.02
5.6	47.86	9.6	86.28	13.6	96.14
5.7	49.36	9.7	86.75	13.7	96.27
5.8	50.83	9.8	87.21	13.8	96.39
5.9	52.28	9.9	87.64	13.9	96.51
6	53.69	10	88.07	14	96.63
6.1	55.07	10.1	88.47	14.1	96.75
6.2	56.43	10.2	88.86	14.2	96.87
6.3	57.75	10.3	89.24	14.3	96.98
6.4	59.04	10.4	89.60	14.4	97.10
6.5	60.31	10.5	89.94	14.5	97.21
6.6	61.54	10.6	90.27	14.6	97.33
6.7	62.75	10.7	90.59	14.7	97.44
6.8	63.93	10.8	90.90	14.8	97.55
6.9	65.08	10.9	91.19	14.9	97.66
7	66.20	11	91.48	15	97.77
7.1	67.29	11.1	91.75	15.1	97.88
7.2	68.35	11.2	92.01	15.2	97.98
7.3	69.39	11.3	92.26	15.3	98.09
7.4	70.40	11.4	92.50	15.4	98.19
7.5	71.38	11.5	92.73	15.5	98.29
7.6	72.33	11.6	92.95	15.6	98.39
7.7	73.26	11.7	93.16	15.7	98.48
7.8	74.16	11.8	93.37	15.8	98.57
7.9	75.03	11.9	93.56	15.9	98.66
8	75.88	12	93.75	16	98.74
8.1	76.70	12.1	93.94	16.1	98.82
8.2	77.50	12.2	94.12	16.2	98.90
8.3	78.27	12.	94.29	16.3	98.97
8.4	79.02	12.4	94.45	16.4	99.03
8.5	79.75	12.5	94.62	16.5	99.09
8.6	80.45	12.6	94.77	16.6	99.14
8.7	81.13	12.7	94.92	16.7	99.19
8.8	81.78	12.8	95.07	16.8	99.22
8.9	82.42	12.9	95.22	16.9	99.25
9	83.03	13	95.36

**Table 6:** New table to convert the maturity score to dental age for girls

Age	Score	Age	Score	Age	Score
girls					
5.1	41.82	9.1	87.01	13.1	97.39
5.2	43.76	9.2	87.54	13.2	97.46
5.3	45.65	9.3	88.05	13.3	97.53
5.4	47.49	9.4	88.54	13.4	97.59
5.5	49.27	9.5	89.02	13.5	97.65
5.6	51.00	9.6	89.47	13.6	97.71
5.7	52.67	9.7	89.91	13.7	97.76
5.8	54.30	9.8	90.33	13.8	97.81
5.9	55.87	9.9	90.74	13.9	97.85
6	57.40	10	91.13	14	97.90
6.1	58.89	10.1	91.50	14.1	97.94
6.2	60.33	10.2	91.86	14.2	97.98
6.3	61.72	10.3	92.21	14.3	98.01
6.4	63.07	10.4	92.54	14.4	98.05
6.5	64.38	10.5	92.85	14.5	98.09
6.6	65.65	10.6	93.16	14.6	98.12
6.7	66.88	10.7	93.45	17.7	98.16
6.8	68.08	10.8	93.73	14.8	98.20
6.9	69.23	10.9	9.99	14.9	98.24
7	70.35	11	94.25	15	98.28
7.1	71.44	11.1	94.49	15.1	98.32
7.2	72.48	11.2	94.72	15.2	98.37
7.3	73.50	11.3	94.94	15.3	98.41
7.4	74.48	11.4	95.15	15.4	98.47
7.5	75.44	11.5	95.35	15.5	98.52
7.6	76.36	11.6	95.54	15.6	98.58
7.7	77.25	11.7	95.72	15.7	98.65
7.8	78.11	11.8	95.89	15.8	98.72
7.9	78.95	11.9	96.05	15.9	98.79
8	79.75	12	96.20	16	98.88
8.1	80.53	12.1	96.34	16.1	98.97
8.2	81.29	12.2	96.48	16.2	99.06
8.3	82.02	12.3	96.61	16.3	99.17
8.4	82.72	12.4	96.73	16.4	99.29
8.5	83.40	12.5	96.84	16.5	99.41
8.6	84.06	12.6	96.95	16.6	99.55
8.7	84.69	12.7	97.05	16.7	99.70
8.8	85.30	12.8	97.14	16.8	99.86
8.09	85.89	12.9	97.23	16.9	100.03
9	86.46	13	97.31		

### Comparison between chronological age and modified dental age

The accuracy of the modified method was tested to prove that the modified model is suitable for use in eastern Chinese children. The percentages of CA-DA values within ± 1 year of the modified method were 83.7% for boys and 79.6% for girls (Table 7). In the Demirjian method, the percentage accuracies were 73.9% for boys and 70.2% for girls for the absolute difference values within 1 year. The Willems method presented a better percentage of accuracy for the absolute difference values within 1 year, which were 75.0% for boys and 75.9% for girls. The accuracy was significantly improved compared with those in the Demirjian and Willems methods. Among them, the accuracy rate of the modified method in ages 5 and 6 years most significantly increased compared with that of the Demirjian method, and compared with that of the Willems method, the most obvious increase was in ages 13–16 years.

**Table 7:** Percentages of accuracy for dental age (DA) estimated using the Demirjian and Willems methods for difference values within 1 year from chronological age (CA) for both sexes

Age	Modified method	
	Male	Female
5	33 (82.5%)	43 (93.48%)
6	115 (91.3%)	84(82.35%)
7	94 (90.4%)	88(83.02%)
8	89 (88.1%)	107(95.54%)
9	92 (94.8%)	80(87.91%)
10	100 (92.6%)	76(67.86%)
11	90 (78.9%)	73(63.48%)
12	84 (75.0%)	94(74.02%)
13	81 (74.3%)	74(79.54%)
14	76 (73.8%)	76(78.35%)
15	88 (80.7%)	77 (81.91%)
16	77 (81.9%)	43 (78.18%)
Total	1019 (83.7%)	915(79.6%)

**Fig. 1:**
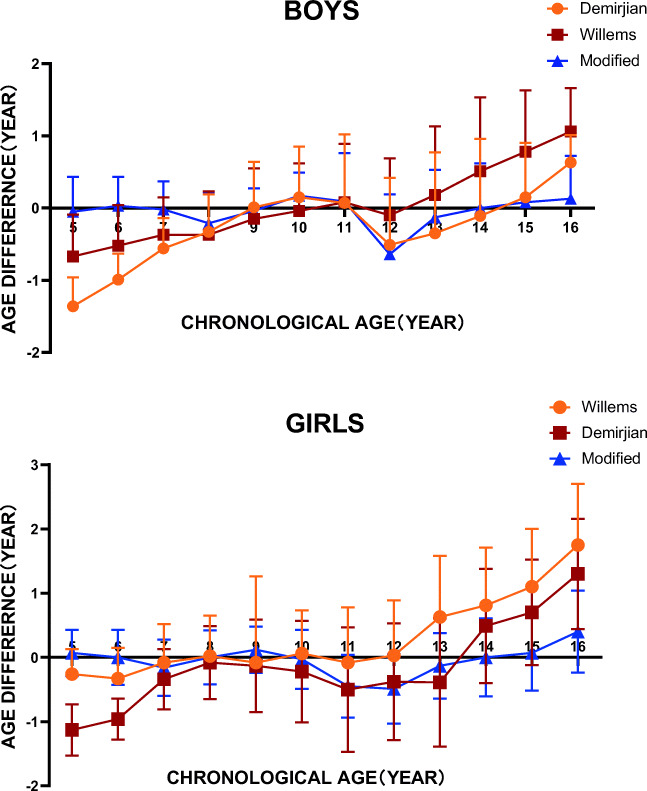
Comparison of dental age between the modified method, the Demirjian method, and the Willems method by gender

The descriptive statistics for the CA-DA is shown in Table 8 and Fig. 1. It resulted in a smaller underestimation compared with CA for both boys (0.04 year) and girls (0.09 year), which showed a high suitability to Chinese children to some extent. The age differences in both sexes were between 0 and 0.5 years, except in boys aged 12 years. The MAE of each age group ranged from 0.31 to 0.88 years, which was quite closer to the CA compared with those in the Demirjian (ranging from 0.36 to 1.36 years) and Willems methods (ranging from 0.47 to 1.68 years).

## Discussion

Accurate age estimation techniques are increasingly needed due to the increasing number of unidentified bodies, as well as age-dependent criminal cases and absence of a valid birth certificate for individuals. In many developed and developing countries, including China, DA estimation of children is widely applied. Ages 14, 16, and 18 years are important limitations in legal and social spheres, which deciding criminal liability is directly responsible by themselves or by the guardian. Ages 5–12 years are mixed dentition period, in which orthodontics and pediatric dentistry treatment are needed.

As is well known, the advantages of clinical methods of DA estimation are based on the fact that tooth development is largely controlled by genes [30, 31]. Among various DA estimation methods, the Demirjian and Williams methods, as the methods using panoramic radiographs to depict the eight stages of tooth mineralization and apical closure, have gained wide attention in different regions because these methods are easy to perform and inexpensive and the subjects are accessible [12, 21]. Considering that dental development is partly regulated by some factors including ethnicity and social environment, the authors speculated that their estimation may be inaccurate in other populations. A series of publications have revealed its inaccuracies [19, 21–23, 25]. Hence, rather than setting a universal standard, a population-specific standard will achieve the most accurate age assessment.

The present study was conducted to establish a formula that is more applicable to Chinese children and supplement the accuracy and applicability of the three methods for dental estimation of children in eastern China. The age range of studied children was 5 to 16 years, and their nationalities are all Han. Applying the Demirjian method, we concluded an overestimation in DA in general (0.21 year for boys and 0.10 year for girls). In each age group, the dental estimation difference values vary from 0.07 to 1.36 years in boys and 0.01 to 1.30 years in girls. The largest difference was in ages 5 and 16 years. The Willems method simplifies the Demirjian method by allowing the estimated age to be calculated directly from the new score adapted from the original maturity score. In our study, for boys, the DA was more overestimated than CA with 0.07 year for boys and 0.24 year for girls, which was similar with those in the Demirjian method. The difference values for each age group were generally low, ranging from 0.04 to 1.06 years in boys and 0.02 to 1.75 years in girls. However, significant underestimation was expressed in older ages, especially in ages 15 and 16 years. Compared with it, the Demirjian method presented more accurate results in both sexes from ages 12 to 16 years. These results are consistent with those of previous studies. Zhai et al. [27] and Wang et al. [25] evaluated 11–18-year-old children and found that the Demirjian method was more accurate.

Studies have shown variations in both dental emergence and formation in different ethnic populations, since people of various nationalities differ in biological maturity [21]. A study compared the biological maturity from the hand-wrist radiographs of Asian, Hispanic, and African-American children and found that Asian and Hispanic children mature earlier than other children [27]. However, to date, there is no suitable formula for Chinese DA estimation. Hence, we analyzed the reasons behind the inaccuracy of the two methods used in Chinese children. The largest difference between the DA and CA of the Demirjian method was noted in lower ages, especially in ages 5 and 6 years, resulting in a significant overestimation. As for the Willems method, the largest difference was noted in ages 13–16 years, generally expressing underestimation. Thus, we, therefore, use polynomial fitting formula to build a new table. We reduced the error by increasing the order, but at the same time, overfitting became more and more serious. To solve this problem, we expanded the sample data to more than 2000. After using the polynomial curve fitting to reevaluate the relationship between the scores and CA, the scores corresponding to lower ages increased in the new table.

In analyzing each age cohort separately in the 2367 samples, the modified method proved to be more accurate than the Demirjian and Willems methods in most age groups (Table 8 and Fig. 1). The lowest mean differences in boys were 0.05, 0.03, 0.02, 0.04, and 0.00 years for ages 5, 6, 7, 9, and 14 years, respectively. Moreover, girls, especially those aged 6, 8, and 14 years, with mean differences of 0.00, had an accurate DA estimation. The MAEs were 0.63 year for boys and 0.64 year for girls, which were both less than those in the two methods. The percentages of accuracy within 1 year sharply increased in lower ages compared with those in the Demirjian method, especially in age of 5 years, from 15.0 to 82.5% for boys and from 34.8 to 93.5% for girls. It also significantly increased in ages 13–16 years compared with those in the Willems method.

**Table 8:** Differences between mean chronological age (CA) and calculated dental age (DA) using the modified method and p-values of the differences in various age groups for both sexes

Age	Sex	Chronological age	Mean (SD)		95% CI of age difference	t statistics	p values	Mae
			Modified dental age (MDA)	Age difference (CA-MDA)				
5.00-5.99	M	5.73 (0.18)	5.78 (0.80)	-0.05 (0.48)	-0.28; 0.19	-0.41	0.684	0.57
	F	5.67 (0.26)	5.60 (0.50)	0.07 (0.36)	-0.07; 0.21	1.04	0.828	0.31
6.00-6.99	M	6.47 (0.29)	6.44 (0.67)	0.03 (0.40)	-0.06; 0.14	0.69	0.492	0.41
	F	6.48 (0.26)	6.48 (0.85)	0.00 (0.43)	-0.15; 0.14	-0.03	0.977	0.63
7.00-7.99	M	7.50 (0.32)	7.52 (0.78)	-0.02 (0.39)	-0.16; 0.10	-0.41	0.684	0.54
	F	7.59 (0.28)	7.75 (0.76)	-0.16 (0.44)	-0.30; -0.02	-2.26	0.026	0.61
8.00-8.99	M	8.39 (0.26)	8.60 (0.66)	-0.21 (0.42)	-0.33; -0.08	-3.22	0.001	0.53
	F	8.48 (0.29)	8.48 (0.62)	0.00 (0.42)	-0.10; 0.11	0.05	0.961	0.37
9.00-9.99	M	9.53 (0.29)	9.57 (0.55)	-0.04 (0.31)	-0.15; 0.08	-0.64	0.524	0.46
	F	9.45 (0.29)	9.33 (0.65)	0.12 (0.36)	-0.01; 0.25	1.84	0.068	0.53
10.00-10.99	M	10.50 (0.30)	10.33 (0.68)	0.17 (0.32)	-0.05; 0.28	2.91	0.004	0.52
	F	10.49 (0.29)	10.52 (0.98)	-0.03 (0.46)	-0.19; 0.13	-0.34	0.734	0.72
11.00-11.99	M	11.42 (0.25)	11.33 (1.04)	0.09 (0.67)	-0.09; 0.26	0.95	0.342	0.69
	F	11.55 (0.30)	12.00 (0.95)	-0.45 (0.49)	-0.61; -0.29	-5.60	<0.001	0.84
12.00-12.99	M	12.37 (0.28)	13.01 (1.04)	-0.64 (0.83)	-0.83; -0.45	-6.62	<0.001	0.88
	F	12.48 (0.29)	12.97 (0.72)	-0.49 (0.54)	-0.63; -0.36	-7.25	<0.001	0.73
13.00-13.99	M	13.48 (0.30)	13.61 (1.06)	-0.13 (0.66)	-0.33; 0.06	-1.34	0.182	0.82
	F	13.43 (0.25)	13.56 (0.85)	-0.13 (0.51)	-0.30; 0.05	-1.45	0.149	0.66
14.00-14.99	M	14.48 (0.30)	14.48 (1.02)	0.00 (0.62)	-0.20; 0.18	-0.12	0.904	0.75
	F	14.57 (0.30)	14.57 (0.93)	0.00 (0.61)	-0.19; 0.18	-0.08	0.940	0.69
15.00-15.99	M	15.48 (0.29)	15.40 (1.05)	0.08 (0.68)	-0.10; 0.27	0.90	0.372	0.69
	F	15.37 (0.23)	15.30 (0.90)	0.07 (0.59)	-0.11; 0.26	0.76	0.448	0.69
16.00-16.99	M	16.40 (0.27)	16.27 (0.94)	0.13 (0.59)	-0.04; 0.32	1.49	0.140	0.69
	F	16.45 (0.24)	16.05 (0.98)	0.40 (0.64)	0.14; 0.64	3.16	0.003	0.77
Total	M	11.20 (3.29)	11.24 (3.38)	-0.04 (1.07)	-0.10; 0.00	-2.09	0.037	0.63
	F	10.99 (3.12)	11.08 (3.22)	-0.09 (1.03)	-0.14; -0.04	-3.77	<0.001	0.64

The estimated age difference (CA-DA) using the modified method was − 0.04 year for boys and − 0.09 year for girls, and it estimated the age of 83.7% of boys and 79.6% of girls within ± 1 year range, showing it to be more accurate than Demirjian method and Willems method. For example, Wang et al. showed that the Demirjian method was found to be more accurate compared with the Willems method for estimation of the ages of children from eastern China, with the mean underestimations of 0.66 year for boys and 0.62 year for girls [25]. Yang et al. found that, in 8–16-year-old southern Chinese children, the Willems method underestimated DA by 0.03 year for girls and overestimated it by 0.03 year for boys, and the Demirjian method underestimated DA by 0.54 year for girls and 0.44 year for boys [25]. Jayaraman J et al. reported that for the southern Chinese specific dental reference datasets, the age difference was − 0.02 for both gender and 80% of subjects were within ± 12 months range, which accuracy is basically the same as the modified method. However, their number of samples is 254, which is relatively small, so the error is more likely to exist [32].

However, in either the Demirjian method or the modified method, the score intervals corresponding to the age at an interval of 0.1 year in older ages were quite close. Therefore, the inaccurate estimation of the root closure degree of any mandibular left tooth will lead to a large error of DA. It is suggested to subdivide the stages of tooth development and design a software to precisely evaluate the stages to reduce the error of the score.

According to the score system in the Demirjian method, if the root apices of the seven left mandibular teeth are all closed, the sum of the maturity score is 100 for boys and 98.4 for girls, which both correspond to the age of 16 years. It must be determined whether the Demirjian and Willems methods both have estimation errors in ages 16–18 years. Based on the Demirjian method, the third molar can be taken into consideration in deciding if an unidentified person has reached 18 years of age. Concurrently, cone beam computed tomography can be used to establish a 3D model and increase the measurement accuracy [20, 33].

## Conclusion

The study was conducted to establish a modified dental age estimation suitable for eastern Chinese children. The Demirjian method was found to be unreliable in lower ages (5–11 years), while the Willems method was unreliable in older ages (12–16 years). Neither the Demirjian method nor the Willems method was accurate. Thus, PCFM was set to convert the maturity score in the Demirjian method to the DA of eastern Chinese children. A further study should analyze the applicability of the new table in other parts of China.
